# MISREMEMBERING SOLITUDE IN OLDER AGE: AGE AND CULTURE SHAPE RECALL ACCURACY FOR LONELINESS AND HAPPINESS IN SOLITUDE

**DOI:** 10.1093/geroni/igae098.4241

**Published:** 2024-12-31

**Authors:** Jennifer Lay, Yuen Wan HO, Dwight Tse, Da Jiang

**Affiliations:** University of Exeter, Exeter, England, United Kingdom; Lingnan University, Hong Kong, Guangdong, China; University of Strathclyde, Glasgow, Scotland, United Kingdom; Education University of Hong Kong, Hong Kong, China (People’s Republic)

## Abstract

Recalling past affective experiences is key to guiding behaviour – including social and solitary activities – in ways that maximise wellbeing. However, affect recall is not always accurate. Previous research suggests that older (vs. younger) adults show a positivity effect in memory, depending on whether the stimuli are culturally meaningful. It is unclear, however, whether this age-related positivity effect applies to recalling experiences of solitude (time without social contact), which become increasingly prevalent in older adulthood. An age-stratified sample of adults aged 18-81 (M age = 40.8 years, 60.8% female) in the United Kingdom (N = 214) and Hong Kong (N = 220) reported their current affective experiences and social situations 5 times per day over 7 days using a smartphone app. Participants then recalled how they felt on average over the 7-day period at times when they were in solitude. Models controlling for peak and recent affect, overall time in solitude, and independent/interdependent self-construal showed that whereas younger adults tended to retrospectively overestimate how lonely they felt in solitude, this effect reversed with age such that older adults tended to underestimate their loneliness in solitude. Moreover, whereas UK older adults tended to retrospectively overestimate their happiness in solitude, HK older adults tended to underestimate their happiness in solitude. Findings suggest individuals misremember their solitude experiences in ways that align with age-normative and cultural attitudes to solitude, and that there is an age-related positivity effect which is shaped by culture. We discuss implications for loneliness and wellbeing in older adulthood.

